# Supplementary material for: Transcriptome Analysis of Eggplant under Salt Stress: AP2/ERF Transcription Factor SmERF1 Acts as a Positive Regulator of Salt Stress
Source: Plants (Basel). 2022 Aug 25;11(17):2205. doi: 10.3390/plants11172205 (PMC9460861; doi:10.3390/plants11172205)
Supplement: Supplementary file 1 [file plants-11-02205-s001.zip › plants-1826284-supplementary/Figure S1.pdf]

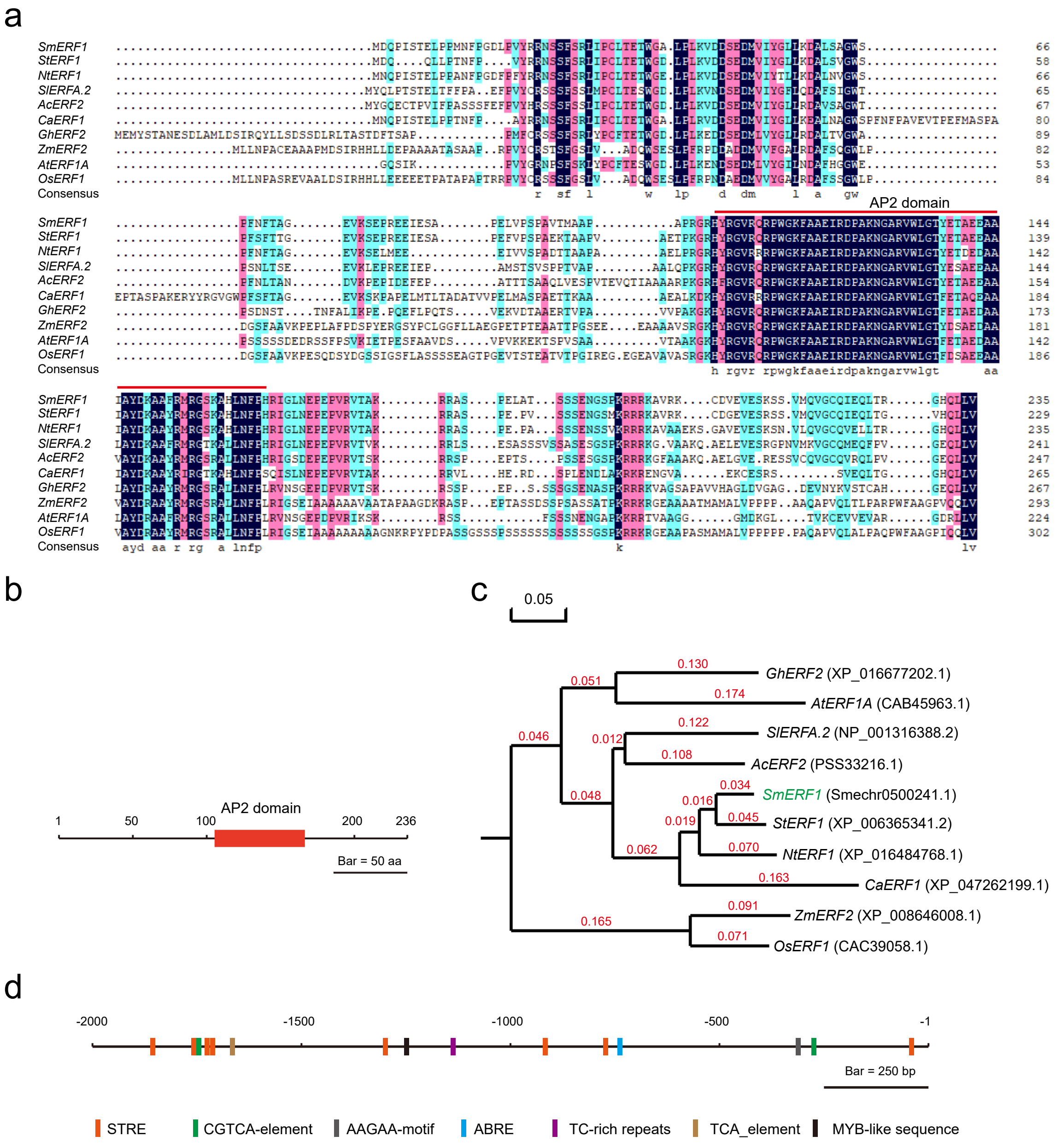
a
SmERF1
StERF1
NtERF1
SlERFA.2
AcERF2
CaERF1
GhERF2
ZmERF2
AtERF1A
OsERF1
Consensus
66
58
66
65
67
80
89
82
53
84
AP2 domain
144
139
142
144
154
184
173
181
142
186
235
229
235
241
247
265
267
293
224
302
b
AP2 domain
1
50
100
200
236
Bar = 50 aa
c
0.05
GhERF2 (XP_016677202.1)
AtERF1A (CAB45963.1)
SlERFA.2 (NP_001316388.2)
AcERF2 (PSS33216.1)
SmERF1 (Smechr0500241.1)
StERF1 (XP_006365341.2)
NtERF1 (XP_016484768.1)
CaERF1 (XP_047262199.1)
ZmERF2 (XP_008646008.1)
OsERF1 (CAC39058.1)
0.130
0.051
0.174
0.046
0.122
0.012
0.108
0.034
0.016
0.045
0.048
0.019
0.070
0.062
0.163
0.091
0.165
0.071
d
-2000
-1500
-1000
-500
-1
Bar = 250 bp
STRE
CGTCA-element
AAGAA-motif
ABRE
TC-rich repeats
TCA_element
MYB-like sequence
