# Supplementary material for: Transcriptome Analysis of Eggplant under Salt Stress: AP2/ERF Transcription Factor SmERF1 Acts as a Positive Regulator of Salt Stress
Source: Plants (Basel). 2022 Aug 25;11(17):2205. doi: 10.3390/plants11172205 (PMC9460861; doi:10.3390/plants11172205)
Supplement: Supplementary file 1 [file plants-11-02205-s001.zip › plants-1826284-supplementary/Supplemental Figure lenged.pdf]

Transcriptome Analysis of Eggplant under Salt Stress: AP2/ERF Transcription Factor *SmERF1* Acts as a Positive Regulator of Salt Stress

Figure S1

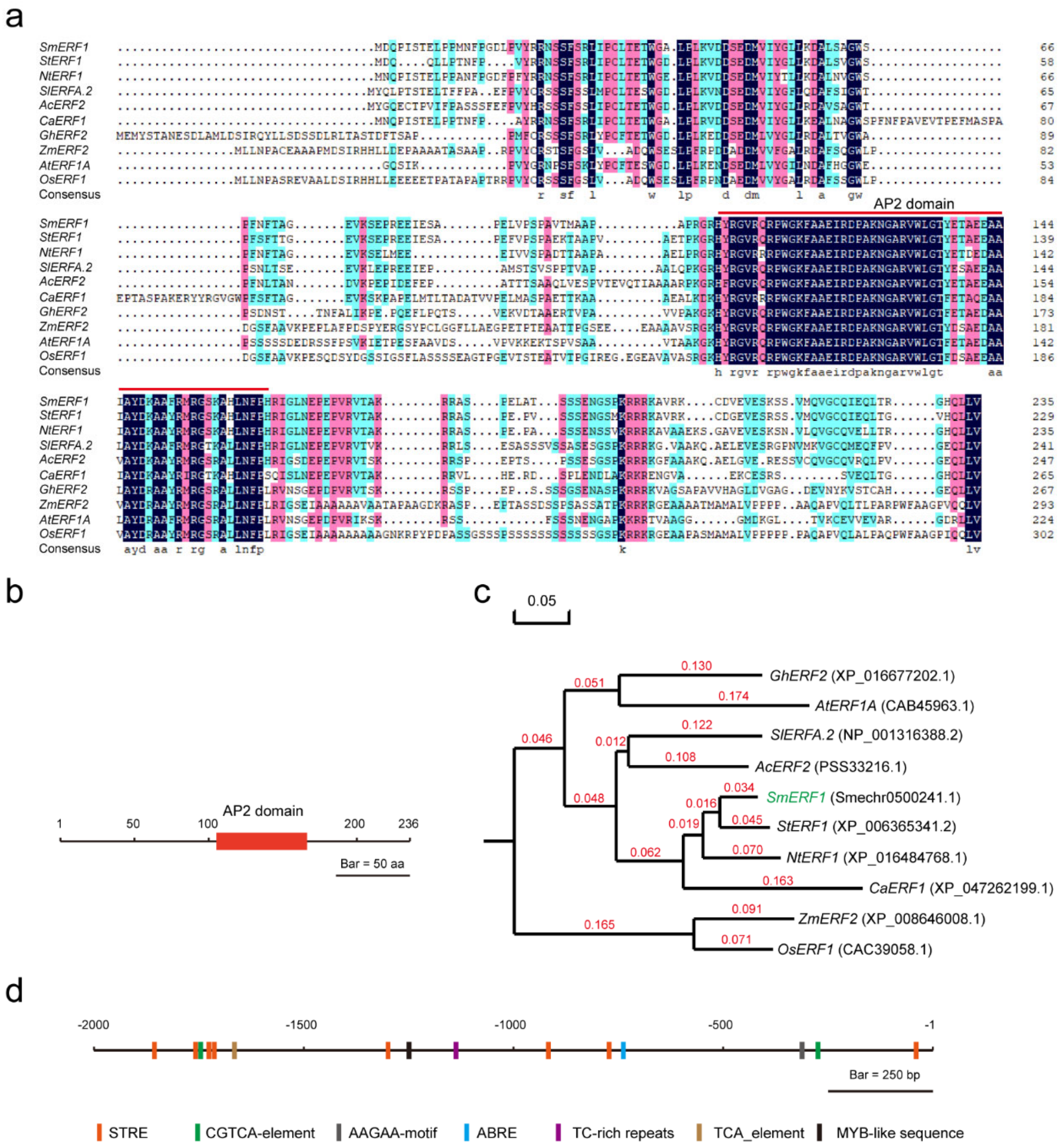

Figure S1. Sequence structure and comparison of *SmERF1*. (**a**) Multiple comparison of *SmERF1* amino acid sequence with its homologs in other plant species using DNAMAN 7.0 (Lynnon Biosoft). *StERF1* (*Solanum tuberosum ERF1*, XP_006365341.2), *NtERF1* (*Nicotiana tabacum ERF1*, XP_016484768.1), *SlERFA.2* (*Solanum lycopersicum ERFA.2*, NP_001316388.2), *AcERF2* (*Actinidia chinensis* var. *chinensis ERF2*, PSS33216.1), *CaERF1* (*Capsicum annuum ERF1*, XP_047262199.1), *GhERF2* (*Gossypium hirsutum ERF2*,

XP_016677202.1), *ZmERF2* (*Zea mays ERF2*, XP_008646008.1), *AtERF1A* (*Arabidopsis thaliana ERF1A*, CAB45963.1), and *OsERF1* (*Oryza sativa ERF1*, CAC39058.1). Wathet blue shading indicates 50% to 75% identity, solferino shading indicates 75% to 100% identity, and black shading indicates 100% identity. The red line denotes the location of AP2 domain. (**b**) Schematic diagram of *SmERF1* amino acid sequence harboring a conserved AP2 domain. The red box indicates the AP2 domain. aa, amino acid. (**c**) Phylogenetic tree of *SmERF1* and its homologs. Red numbers indicate branch length. (**d**) Diagram of cis-elements within the *SmERF1* promoter. The main cis-elements include seven stress response elements (STRE) (orange boxes), two methyl jasmonate response elements (CGTCA-element) (green boxes), one cis-element involving abscisic acid response (AAGAA-motif) (gray box), one abscisic acid responsiveness element (ABRE) (blue box), one defense and stress responsiveness element (TC-rich repeats) (purple box), one salicylic acid responsiveness element (TCA-element) (brown box), and one MYB transcription factor binding site (MYB-like sequence) (black box).

Figure S2

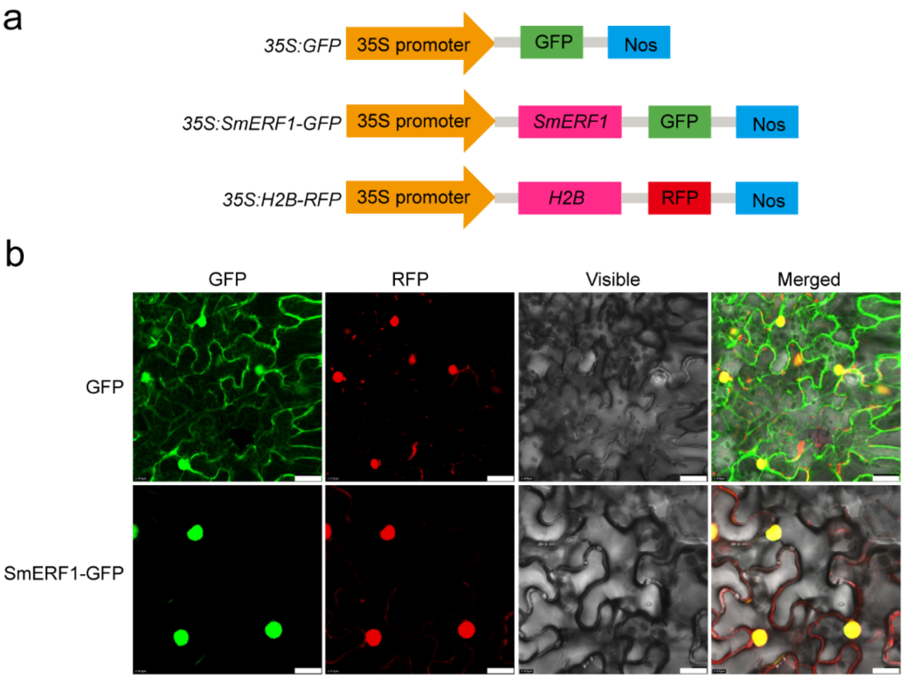

Figure S2. Subcellular localization of SmERF1. (**a**) Schematic diagram of pBinGFP2 and recombinant pBinGFP2-*SmERF1* vector. (**b**) Subcellular localization of SmERF1-GFP or GFP in epidermal cells of *Nicotiana bemthamiana* leaves at 48 h post-infiltration observed under laser scanning confocal microscope. Scale bar = 25 μm. GFP, green fluorescence protein; RFP, rad fluorescence protein.
