# Supplementary material for: Transcriptome Analysis of Eggplant under Salt Stress: AP2/ERF Transcription Factor SmERF1 Acts as a Positive Regulator of Salt Stress
Source: Plants (Basel). 2022 Aug 25;11(17):2205. doi: 10.3390/plants11172205 (PMC9460861; doi:10.3390/plants11172205)
Supplement: Supplementary file 1 [file plants-11-02205-s001.zip › plants-1826284-supplementary/Table S5.pdf]

Table S5. Primers used in this study

Primers used for qRT-PCR assay			
Gene name	Gene ID	Forward primer (5'→3')	Reverse primer (5'→3')
SmActin	Smechr1100649	TTCCCTTCTTCCCACTAAAGC	AATCTCTCCCTTCTTTCATAGCC
SmERF1	Smechr0500241	CTAGCTCGTCGGAAAACGGT	ATAGCTGATGGCCACGTGTC
SmMAPKKK1	Smechr0701976	TGCTTACTCGCCAGTTTCCT	TCAACCGAAAGGGTATCAGG
SmWRKY20	Smechr0701744	CTGTTGTGTCAGCTGGTGGT	CTTCGGAGGTGGACTCATGT
SmCDPK32	Smechr1002846	AAACGAGGAGCACCTGAAGA	TTCTTCGCTGTTGGTTTCAA
SmCNGC17	Smechr0700014	ATCTTGCAGCACTGGAATCA	TAGGGGACGGATTTGAGCTA
SmHSP90-5	Smechr1001880	GAGATGATGGCAATGGCTCT	TCTTGGACTTCAGCGGAGTT
SmRIN1	Smechr0800184	TCACCGAGCTGCATAATCTG	CTTCACCTTCCACAGCTTGC
SmCDPK11	Smechr1200644	CTCTTGGGCCCAGTGATAGT	AGCCAACTAGGTGGGCTTCT
SmGSTU10	Smechr0303072	GGGTGACCACTCTGCTTGAG	TGAAACGTAAAGCCATTTTGC
SmABA8'HO3	Smechr0401604	CCTGGTCCGAAGTCAACAAT	TTGATTTTGCACCCCTCAAT
SmDHN1	Smechr0200704	CAACTCATGCTGCACAAACA	CAATTCATGCCAGGGATCTT
SmDHNX1	Smechr0200676	TCCTGCTCCTACTCCTGCTC	GCATCCCAGTGATCTTTTCC
SmTAS14	Smechr0202316	GGACAGCAGGAAGGTGGATA	TCATTCCAGGGATCTTGTCC
Primers used for vector construction			
SmERF1-TOE	Smechr0500241	CTGTACAAGGGTACCCCC GGGATGGATCAACCAATTT CTACCGA	AGAGGATCCGTCGACCCCGG GTTAACTGACCAATAGCTGAT GGCC
SmERF1-VIGS	Smechr0500241	GGGGACAAGTTTGTACAAA AAAGCAGGCTTCATGGATC AACCAATTTCTAC	GGGGACCACTTTGTACAAGA AAGCTGGGTCCGGAGCTGCC ATAGTCACCG

SmERF1-TOE means the primers used for vector construction of *SmERF1* transient overexpression;

SmERF1-VIGS means the primers used for vector construction of *SmERF1* silencing.
