# Supplementary figures and images for: Transcriptome Analysis of Eggplant under Salt Stress: AP2/ERF Transcription Factor SmERF1 Acts as a Positive Regulator of Salt Stress
Source: Plants (Basel). 2022 Aug 25;11(17):2205. doi: 10.3390/plants11172205 (PMC9460861; doi:10.3390/plants11172205)

a

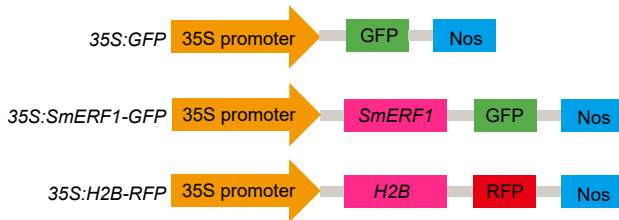

b

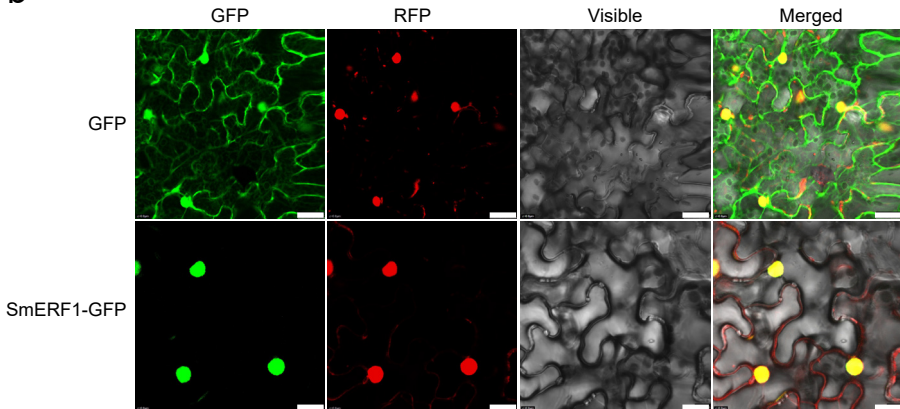

Supplement: Supplementary file 1 [file plants-11-02205-s001.zip › plants-1826284-supplementary/Figure S2.pdf]
